# Exercise-Induced Coronary Remodeling and the Atherosclerotic Paradox in Endurance Athletes: Toward a Unified Mechanobiological Framework

**DOI:** 10.3390/jfmk11030265

**Published:** 2026-07-04

**Authors:** Nardi Tetaj, Andrea Segreti, Michele Pelullo, Camilla Rossi, Alberto Spagnolo, Virginia Ligorio, Aurora Ferro, Antonio Emanuele Lentini, Teresa Trunfio, Martina Ciancio, Chiara Fossati, Fabio Pigozzi, Francesco Grigioni

**Affiliations:** 1 Cardiology Unit, Campus Bio-Medico Hospital University, 00128 Rome, Italy; nardi.tetaj@unicampus.it (N.T.); michele.pelullo@unicampus.it (M.P.); camilla.rossi@unicampus.it (C.R.); alberto.spagnolo@unicampus.it (A.S.); virginia.ligorio@unicampus.it (V.L.); aurora.ferro@unicampus.it (A.F.); a.lentini@unicampus.it (A.E.L.); teresa.trunfio@unicampus.it (T.T.); martina.ciancio@unicampus.it (M.C.); f.grigioni@policlinicocampus.it (F.G.); 2 Research Unit of Cardiovascular Science, Department of Medicine and Surgery, Campus Bio-Medico Hospital University, 00128 Rome, Italy; 3 Department of Movement, Human and Health Sciences, University of Rome “Foro Italico”, 00135 Rome, Italy; chiara.fossati@uniroma4.it (C.F.); fabio.pigozzi@uniroma4.it (F.P.)

**Keywords:** coronary artery disease, coronary atherosclerosis, endurance exercise, master athletes, coronary artery calcium, CCTA, plaque burden, exercise-induced remodeling

## Abstract

Regular endurance exercise is consistently associated with lower cardiovascular mortality, a favorable cardiometabolic profile, and superior cardiorespiratory fitness. However, coronary imaging studies in master endurance athletes have raised a clinically relevant paradox: despite a low burden of conventional risk factors, some athletes—particularly older men with high lifetime exercise exposure—show a greater prevalence of coronary artery calcium and subclinical coronary plaque than sedentary or less active controls. This observation has challenged the long-standing assumption that high-volume endurance exercise is uniformly protective against coronary artery disease. A binary interpretation of this literature is inadequate. Coronary flow reserve and ischemic threshold may remain adequate in some athletes, although this concept is supported by limited functional and outcome data. Based on experimental vascular biology and indirect human evidence, repetitive high-flow states during endurance exercise generate sustained laminar shear stress, cyclic wall strain, and marked increases in coronary blood flow, thereby activating endothelial mechanotransduction pathways and influencing vascular smooth muscle cell behavior, extracellular matrix remodeling, and calcification biology. These adaptations may culminate in positive arterial remodeling, luminal enlargement, and, in some individuals, a predominantly calcified plaque phenotype. Importantly, structural remodeling does not necessarily equate to functional impairment. In selected athletes, when outward remodeling and endothelial responsiveness are preserved, coronary flow reserve and ischemic threshold may remain adequate, although this concept remains supported by limited functional and outcome data. This narrative review integrates the clinical imaging literature with current concepts in vascular mechanobiology to propose that coronary remodeling in endurance athletes exists along an adaptive–maladaptive continuum shaped by cumulative exercise load, aging, sex, conventional risk factors, and biological susceptibility. This framework may help clinicians interpret CAC/CCTA findings in athletes more appropriately and avoid equating plaque burden with equivalent functional or prognostic significance.

## 1. Introduction

Endurance exercise is universally associated with reduced cardiovascular risk, improved metabolic profile, enhanced endothelial function, and increased cardiorespiratory fitness [1]. Yet, an apparent paradox has emerged in master endurance athletes: despite favorable traditional risk factors and high aerobic capacity, several imaging studies report a greater prevalence of coronary artery calcification compared with sedentary controls [2,3]. This observation has fueled an ongoing debate—does lifelong endurance training confer protection or promote coronary atherosclerosis? The binary framing of this question, however, may oversimplify a far more complex biological process.

This apparent contradiction has generated the concept of an atherosclerotic paradox in endurance athletes.

The concept that endurance exercise may coexist with coronary atherosclerosis is not new. Early work by Möhlenkamp and colleagues drew attention to the paradox of coronary atherosclerosis in apparently healthy marathon runners and subsequently established the prognostic relevance of coronary calcium burden in veteran male runners [4,5,6]. In parallel, vascular physiology studies and reviews, including the work by Green and colleagues on the concept of an “athlete’s artery”, emphasized that athletic training can induce structural and functional vascular adaptation beyond the myocardium [7,8]. However, the paradox is probably not resolved by asking whether exercise is either protective or harmful [9,10]. That framing is too simplistic for a vascular system that is inherently load-responsive. Coronary arteries are not passive conduits. They are biologically active, mechanically sensitive structures that continually adapt to changing hemodynamic conditions. From this perspective, endurance exercise represents a chronic vascular stimulus capable of inducing both physiological remodeling and, in a subset of susceptible individuals, structural changes that overlap with subclinical atherosclerosis [11]. The central challenge, therefore, is not simply to determine whether athletes have more calcification, but to understand what that calcification means. In sedentary populations, CAC is an established marker of atherosclerotic burden and future risk [12,13,14]. Importantly, this principle should not be dismissed in athletes. Although plaque morphology and functional capacity may modify clinical interpretation, CAC remains a robust marker of total coronary atherosclerotic burden, and current evidence does not establish that elevated CAC in athletes carries fundamentally different prognostic implications than in non-athletic populations [15,16,17].

The aim of this narrative review is to integrate mechanical loading, endothelial mechanotransduction, structural remodeling, plaque phenotype, and functional performance into a unified coronary-specific framework. We begin by acknowledging the historical perspective on vascular adaptation in athletes and coronary atherosclerosis in endurance populations, including foundational work by Möhlenkamp et al. and Green et al. Building on this literature, we examine coronary remodeling as a load-conditioned biological response in endurance athletes and propose a mechanobiological continuum that may help distinguish adaptive vascular remodeling from pathological atherosclerosis. This framework should be interpreted as a conceptual model intended to organize selected clinical and mechanistic evidence, rather than as a proven causal pathway. While clinical imaging studies demonstrate altered CAC and plaque phenotypes in selected endurance-athlete cohorts, several mechanistic links between exercise-induced hemodynamic stress, endothelial mechanotransduction, inflammation, and calcific plaque transformation remain inferred from experimental vascular biology rather than directly proven in endurance athletes.

## 2. Literature Search Strategy

A selective narrative literature search was conducted across PubMed/MEDLINE, Scopus, Web of Science, Embase, Cochrane Library, and Google Scholar to identify relevant studies published from January 2000 up to the date of the final revised search. The search focused on studies evaluating the relationship between long-term exercise training, particularly endurance exercise, and coronary atherosclerosis, with special attention to cohorts of master athletes, marathon runners, cyclists, and other highly trained or lifelong endurance athletes assessed by coronary artery calcium scoring, coronary computed tomography angiography, or related imaging modalities. Search terms combined keywords such as athletes, endurance exercise, master athletes, marathon runners, coronary atherosclerosis, coronary artery calcium, CAC, coronary computed tomography angiography, CCTA, plaque, coronary plaque, coronary artery disease, training load, and exercise intensity.

This review was conceived as a narrative review rather than as a systematic review or scoping review. Therefore, no PRISMA or PRISMA-ScR framework was applied, no protocol was registered, no formal dual-review screening process was performed, no PRISMA flow diagram was generated, and no structured risk-of-bias assessment was conducted. Studies were selected and discussed according to their clinical relevance, methodological quality, imaging detail, mechanistic relevance, and contribution to the evolving concept of exercise-associated coronary remodeling and plaque phenotype in athletes. As a result, the review remains vulnerable to citation selection bias and does not provide a comprehensive or quantitative estimate of prevalence, prognosis, or causality.

We included relevant English-language studies involving humans, with emphasis on cross-sectional cohorts, prospective and retrospective observational studies, longitudinal imaging studies, and major review or consensus papers that provided important contextual interpretation of the field. Conference proceedings, corrections, early access articles without final data, news items, book chapters, retractions, reprints, biographical items, book reviews, meeting abstracts, editorial materials, and letters were excluded unless considered essential for historical or conceptual context. Earlier foundational publications before 2000 were included selectively when directly relevant to the historical development of the field. Accordingly, the conclusions of this review should be interpreted as based on selected evidence rather than as a systematic synthesis of all available literature.

## 3. Current Evidence of Coronary Artery Disease in Athletes

The current literature on the relationship between long-term exercise training and coronary atherosclerosis is complex and does not support a single uniform pattern across all athletic populations. Rather, it suggests that the coronary phenotype of athletes is heterogeneous and influenced by sex, age, comparator selection, exercise exposure, and imaging modality. The strongest and most consistent signal has emerged in middle-aged and older male endurance athletes, in whom several studies have demonstrated a higher burden of subclinical atherosclerosis than expected from conventional risk scores.

### 3.1. Athletes-Versus-Control Studies

The first landmark report was the study by Möhlenkamp et al. [4], which compared 108 apparently healthy male marathon runners aged 50 years or older with age-matched and risk-factor-matched controls from the Heinz Nixdorf Recall Study [6]. Despite a substantially lower Framingham risk score (FRS), marathon runners had a CAC burden like age-matched controls and higher than risk-factor-matched controls. In that cohort, 36.1% of runners had CAC ≥ 100, and higher CAC burden was associated with myocardial late gadolinium enhancement and worse event-free survival on follow-up. This study established the important concept that some veteran endurance athletes may carry atherosclerotic burden that appears disproportionate to their otherwise favorable conventional risk profile. The main comparative studies are summarized in Table 1.

Using coronary CT angiography rather than CAC alone, Schwartz et al. then showed that long-term male marathon runners had significantly greater total, calcified, and non-calcified plaque volume than sedentary controls, even though plaque prevalence itself was not significantly different [18]. This was a key step in the field because it shifted attention away from calcium burden alone and toward the broader concept of plaque burden and plaque composition.

A particularly influential low-risk comparison study was that of Merghani et al., who examined 152 master endurance athletes and 92 controls with similarly low Framingham risk profiles [19]. In this highly selected cohort, most athletes and controls still had CAC of zero, but important sex-specific differences emerged. Male athletes had more plaques than sedentary male controls, and only male athletes showed CAC ≥ 300 or luminal stenosis ≥ 50%. However, plaques in male athletes were predominantly calcific, whereas sedentary men more often had mixed plaques, raising the hypothesis that exercise-related plaque may differ phenotypically from plaque in less active individuals [19].

That possible sex difference was reinforced by Roberts et al., who compared long-term female marathon runners with sedentary controls and found that women marathoners had lower plaque prevalence and lower calcified plaque volume, with no significant increase in non-calcified plaque or stenosis severity [20]. Although the control group had a less favorable risk profile and the sample was small, this study suggested that the excess coronary atherosclerosis signal observed in male endurance athletes should not automatically be extrapolated to women.

More recently, the Master@Heart study by De Bosscher et al. provided one of the most rigorous modern datasets in the field [21]. This multicenter study compared lifelong endurance athletes, late-onset athletes, and non-athletic controls, all men with a very low cardiovascular risk profile by design. Unlike earlier studies that supported a more benign calcified-plaque narrative, Master@Heart showed that lifelong athletes had more plaques overall, including proximal, calcified, mixed, and non-calcified plaques, than controls. The prevalence of CAC >100 was also numerically highest in lifelong athletes. This study therefore challenged the reassuring notion that athlete plaque is simply “more calcified and thus safer,” and suggested that very high lifelong endurance exposure may be associated with a broader and more complex coronary plaque phenotype [21]. Because proximal location and non-calcified or mixed morphology are conventionally associated with higher ischemic risk, these findings argue against assuming that coronary plaque in athletes is uniformly stable or clinically benign. Therefore, the athlete plaque phenotype should be considered heterogeneous.

**Figure 1:**
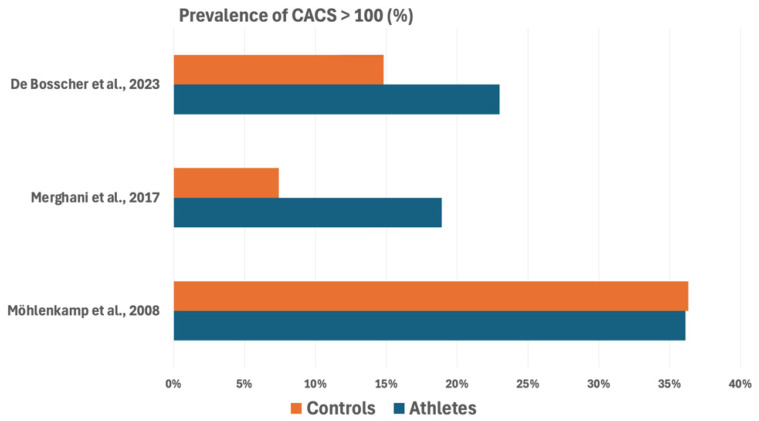
Prevalence of coronary artery calcification scores (CACS) > 100 among main athlete-control studies [4,19,21].

A visual overview of the comparative evidence can be summarized schematically in Figure 1. However, given the substantial heterogeneity across cohorts, direct numerical comparison of CAC thresholds across studies should be interpreted cautiously.

In contrast, Feuchtner et al. reported a more neutral pattern in a retrospective clinical coronary CTA cohort comparing high-exercise individuals with sedentary controls [22]. After matching and adjustment, there were no significant differences in CAC, CAD-RADS severity, obstructive CAD, high-risk plaque, or major adverse cardiovascular events. However, this was not a classic asymptomatic athlete-screening cohort, but rather a symptomatic clinical CTA population, and exercise exposure was self-reported. Accordingly, this study is best interpreted as showing that in a more real-world recreationally active population, approximately 9 h/week of exercise does not necessarily translate into a worse coronary CTA phenotype [22].

Taken together, athlete-versus-control studies suggest that the relationship between endurance exercise and coronary atherosclerosis is not binary. In older male endurance athletes with high cumulative exposure, plaque burden may be greater than expected from conventional risk profiles. Yet the composition of those plaques, and their functional and prognostic implications, remain heterogeneous.

### 3.2. Observational Studies

Athlete-only observational cohorts have added important context by showing that subclinical coronary atherosclerosis is not rare even in the setting of otherwise reassuring sports screening. The athlete-only observational cohorts and longitudinal analyses are summarized in Table 2.

In the MARC study, Braber et al. evaluated 318 asymptomatic middle-aged male sportsmen with normal sports medical evaluation, including resting and exercise ECG [23]. Despite this reassuring profile, 18.9% had occult CAD (8.4% with moderate-to-severe stenosis), defined as CAC ≥ 100 and/or ≥50% stenosis, and 16.4% had CAC ≥ 100. This study showed that routine sports screening does not exclude meaningful subclinical coronary disease in older male athletes.

Within the same MARC framework, Aengevaeren et al. (MARC-1) demonstrated that athletes with the highest lifelong exercise volume had more CAC and more plaque than those in lower exercise-volume strata [24]. Yet among athletes with plaque, the most active individuals more often had calcified-only plaques and fewer mixed plaques, reinforcing the idea that exercise may influence not just plaque quantity but also plaque phenotype.

Other athlete-only cohorts have been broadly consistent. Tsiflikas et al. reported a high prevalence of coronary atherosclerosis in asymptomatic male marathon runners older than 45 years, including proximal plaques and occasional stenoses greater than 50%, despite very favorable conventional risk profiles [25]. Notably, treadmill testing was negative even in the athlete with significant anatomic disease, highlighting the limited sensitivity of standard functional screening for silent coronary plaque.

**Table 2:** Main observational studies assessing the prevalence and clinical significance of coronary atherosclerosis in athletes and highly active individuals.

Study	Study Size	Inclusion Criteria	Exclusion Criteria	Age, Mean or Median	Main Findings	Key Limitations
Möhlenkamp et al., 2014 [5]	108	6.5-year follow-up of the original marathon cohort	History of established CVD, DM, renal failure, and psychiatric disease	63 y at follow-up	Coronary event rates increased stepwise with CAC burden: 12.0% for CAC 100–399, and 21.4% for CAC ≥ 400. Overall event rate in runners was 6.5%. Transient post-race hsTnI elevation did not predict future events, whereas CAC burden and LGE did. All-cause mortality was similar between runners and controls.	Male-only; Small number of coronary events; hsTnI measured shortly after the race, so peak may have been missed.
Tsiflikas et al., 2015 [25]	50	Male marathon runners > 45 y	Known CAD, contrast allergy, GFR < 60 mL/min, hyperthyroidism	52.7 ± 5.9 y	48% had CAD. A total of 34 plaques were detected (17 calcified, 8 mixed, 9 non-calcified). Overall, 24% had proximal plaques, and 10% had CAC > 100: 8% with 100–400, 2% with >400. Treadmill exercise testing was negative for ischemia in all participants.	Male-only; relatively small sample; limited marathon exposure may increase heterogeneity
Braber et al. (MARC), 2016 [23]	318	Asymptomatic high-level sportsmen ≥ 45 y.	Known CAD, contrast allergy, renal impairment	54.7 ± 6.3 years	9.1% had CACS 100–399, and 7.2% had CACS ≥ 400. CCTA showed coronary stenosis ≥ 50% in 5.3%. Overall, occult CAD was detected in 18.9%, defined as CACS ≥ 100 and/or ≥50% luminal stenosis.	Cohort included mixed sport disciplines rather than a pure endurance-only phenotype.
Aengevaeren et al., 2017 (MARC-1) [24]	284	Asymptomatic men ≥ 45 y; competitive or recreational leisure sports, free of known CVD.	Abnormal sports medical examination, known CAD/CVD, renal impairment;	55 ± 7 years	CAC was present in 53% overall. The >2000 MET-min/week group had higher CAC prevalence (68%) and plaque prevalence (77%) than the <1000 MET-min/week group (43% and 56%), with adjusted OR 3.2 for CAC and 3.35 for plaque. Among athletes with plaque, the most active group had fewer mixed plaques and more often only calcified plaques, suggesting a more benign plaque composition despite greater plaque burden.	Male-only, white-only cohort; exercise history self-reported and therefore subject to recall bias.
Gervasi et al., 2019 [26]	167	Age > 35 y; competitive sports for ≥12 months.	Stress-test ECG with bundle branch block.	53.8 ± 10 y	49.7% had a completely negative CCTA. Even among low-SCORE athletes, 17.8% had mild/moderate CA. Male sex, age, and BMI independently predicted CA; hypercholesterolemia, family history, and age predicted multiple plaques.	Limited generalizability because CCTA was used only as a third-level test after PPS abnormalities/symptoms
DeFina et al., 2019 [27]	21,758	Healthy men 40–80 y, from the Cooper Center Longitudinal Study, without prevalent CVD	Follow-up < 1-year, prior MI or stroke.	51.7 ± 8.4 y	Men with ≥3000 MET-min/week were modestly more likely to have prevalent CAC ≥ 100 (adjusted relative risk 1.11, 95% CI 1.03–1.20). However, after 10.4 ± 4.3 years follow-up, high physical activity was not associated with increased all-cause or CVD mortality, even when CAC ≥ 100 was present.	Male-only; predominantly white cohort; physical activity self-reported; not a dedicated athlete cohort; CAC only, without detailed plaque phenotype on CCTA
Lee et al., 2021 (MATCH-40) [28]	65	Age ≥ 40 y and successful completion of the 2018 or 2019 full Manitoba Marathon	Symptoms suggestive of CAD, prior CAD/PCI/CAB.	53 ± 7 y	20% had silent CAD on CCT. Lesions were mainly calcific, with fewer non-calcific and mixed plaques. Among the 13 CCT-positive athletes, all stress echocardiograms were negative for inducible ischemia.	Small sample; SE performed only in CCT-positive athletes; no long-term event follow-up; CAC thresholds not systematically reported
Aengevaeren et al., 2023 (MARC-2) [29]	289	Surviving from MARC-1 at 6-year follow-up	Inherited from MARC-1; in MARC-2, men with PCI during follow-up were excluded from plaque analyses	54 (50–60) y at baseline; 60 (56–66) y at follow-up	CAC prevalence increased from 52% to 71%, and plaque prevalence from 64% to 83% over 6.3 ± 0.5 years. Exercise volume was not associated with CAC or plaque progression. Vigorous-intensity exercise was associated with less CAC progression, whereas very-vigorous-intensity exercise was associated with greater CAC progression and with higher odds of plaque progression, especially calcified plaque progression.	Male-only; almost entirely White cohort; statin use increased over follow-up; longitudinal imaging-surrogate outcomes without direct event adjudication
Berge et al., MARC-2 follow-up, 2023 [30]	289	Men from the MARC-2 study	Established cardiovascular or renal disease at original inclusion.	Median 60 (56–66) y	Median CAC score was 31. Traditional predictors independently associated with greater CAC were older age, higher systolic BP, smoking pack-years, and family history of CAD. Among non-traditional predictors, higher training load, higher serum phosphate, lower daily energy intake, and lower fat percentage of total energy intake were independently associated with higher CAC. Adding these non-traditional variables improved prediction of CAC > 100 and CAC > 400 beyond traditional risk factors alone.	Male cohort; almost entirely White cohort; no plaque-volume/composition data; possible reverse causality for diet/statin-related findings
Pauwels et al., 2026 (Master@Heart wearable-TL analysis) [31]	222	Men 45–70 y from Master@Heart with complete 12 consecutive months of wearable-derived training data and CCTA.	History of CVD; diabetes; hypercholesterolemia; arterial hypertension; BMI > 27.2 kg/m^2^; smoking history.	Median 54 (49–59) y	Compared with the lowest eTRIMP quartile, the highest quartile had greater odds of ≥1 plaque (OR 5.85), ≥1 proximal plaque (OR 3.60), calcified plaque (OR 3.69), partially calcified plaque (OR 5.18), CAC >0 (OR 5.03), and CAC >100 (OR 3.50). (77 lifelong athletes, 98 late-onset athletes, 47 active controls)	Male-only; almost entirely White cohort; 12 months of objective TL may not fully capture lifelong exposure.

Abbreviations: BMI, body mass index; BP, blood pressure; CACS, coronary artery calcium score; CCTA, coronary computed tomography angiography; CAD, coronary artery disease; CAC, coronary artery calcium; CA, coronary atherosclerosis; CVD, cardiovascular disease; DM, diabetes mellitus; GFR, glomerular filtration rate; hsTnI, high-sensitivity troponin I; LGE, late gadolinium enhancement; eTRIMP, Edwards training impulse; MET, metabolic equivalent of task; MI, myocardial infarction; OR, odds ratio; PCI, percutaneous coronary intervention; PPS, pre-participation screening; SE, stress echocardiography; TL, training load.

In more selected clinical settings, the prevalence of coronary disease is understandably higher. Gervasi et al. found that more than 40% of master athletes referred for CCTA because of symptoms or equivocal pre-participation findings had coronary atherosclerosis [26]. These data do not define prevalence in all athletes, but they do support the value of CCTA as a second- or third-line test when clinical suspicion is raised. The large prospective cohort by DeFina et al. broadened the field further by examining more than 21,000 generally healthy men across different physical activity strata [27]. Men with very high activity volumes were modestly more likely to have CAC ≥ 100, but high activity was not associated with increased all-cause or cardiovascular mortality, over about a decade of follow-up, even when clinically relevant CAC was present [27]. This study is particularly important because it helps reconcile the “more CAC, but not necessarily worse prognosis” paradox. The MATCH-40 study by Lee et al. added an especially useful functional dimension by combining CCT with stress echocardiography in marathon runners aged 40 years or older [28]. In this cohort, 20% had silent CAD on CCT, of which 23.1% had moderate-to-severe lesions, but none of those with positive CCT findings had inducible ischemia on stress echocardiography, even when moderate or severe lesions were present. However, this finding should be interpreted cautiously because the cohort was small, stress echocardiography was performed only in CCT-positive athletes, and long-term event data were unavailable.

**Figure 2:**
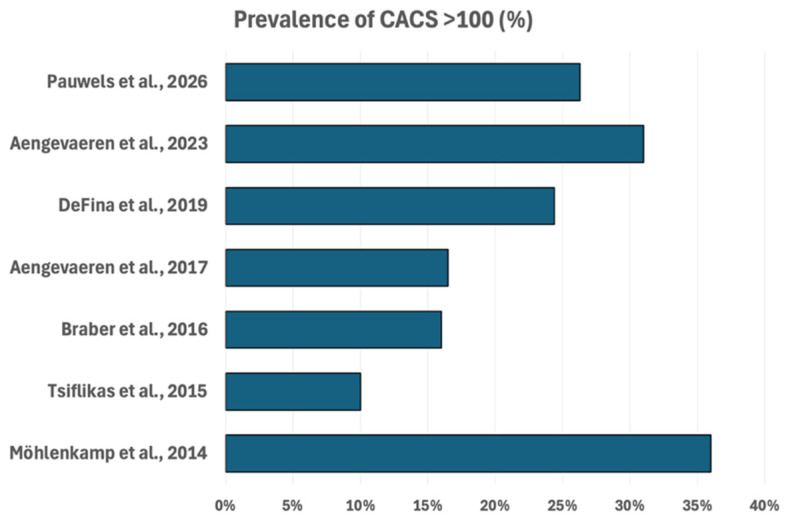
Prevalence of coronary artery calcification scores (CACS) > 100 among main observational studies [5,23,24,25,27,29,31].

Longitudinal observational data have also helped refine interpretation of these cross-sectional findings. In the prognostic follow-up of the original marathon cohort over approximately 6.5 years, by Möhlenkamp et al., coronary event rates increased stepwise with higher baseline CAC burden, whereas transient post-marathon troponin elevation did not predict outcome [4,5]. This suggests that among the biological signals observed in endurance athletes, subclinical coronary atherosclerosis burden carries greater long-term prognostic relevance than transient biomarker release after exercise. More recently, in MARC-2, Aengevaeren et al. showed that over approximately 6.3 years, CAC prevalence increased from 52% to 71% and plaque prevalence from 64% to 83% [29]. This study is particularly important because it is a longitudinal follow-up of the MARC cohort [24], and directly examines progression of CAC and plaque over time, rather than just cross-sectional prevalence. Interestingly, exercise volume during follow-up was not associated with progression, whereas exercise intensity appeared more informative: vigorous exercise was associated with less CAC progression, while very vigorous exercise was associated with greater CAC and calcified plaque progression [29]. The subsequent Berge et al. analysis of the same MARC-2 cohort further showed that, in aging male athletes, traditional risk factors such as age, systolic blood pressure, smoking exposure, and family history remain important predictors of CAC, but that non-traditional variables such as training load, serum phosphate, and diet-related measures add incremental predictive value [30]. This is relevant because it suggests that conventional cardiovascular risk models may incompletely explain coronary atherosclerosis in athletes. A particularly important methodological advance came from Pauwels et al., a secondary analysis of the Master@Heart cohort using 12 months of wearable-derived objective training data [21,31]. In this study, higher objective training duration and higher Edwards training impulse score (eTRIMP) were independently associated with more plaques, more CAC > 0, and more CAC > 100, whereas self-reported MET-based exercise metrics were less informative. Notably, high training duration, especially when combined with cumulative high-intensity effort, appeared more consistently associated with subclinical CAD than intensity alone [31]. These data suggest that future studies of exercise-related CAD may benefit substantially from objective wearable-based exposure assessment rather than relying solely on retrospective questionnaires. The observational literature is therefore better summarized through study-level methodological characteristics rather than through direct visual comparison of CAC prevalence (Figure 2).

Taken together, the evidence summarized in Table 1 and Table 2 supports several broad conclusions. First, long-term endurance exercise does not confer complete protection from coronary atherosclerosis, particularly in middle-aged and older men. Second, the athlete plaque phenotype is likely heterogeneous: some cohorts suggest a predominance of calcified plaque, whereas others, especially Master@Heart [21,31], also report more mixed, non-calcified, and proximal plaques. Third, greater coronary plaque burden or CAC in athletes does not invariably imply demonstrable ischemia in the limited cohorts studied, although the evidence base remains small and does not justify assuming a preserved ischemic threshold in all athletes, as shown by MATCH-40 and DeFina [27,28]. Finally, the effect of exercise exposure appears to depend not only on whether someone is “active,” but on the dose, duration, intensity, and probably also the lifetime pattern of endurance training. Overall, the evidence suggests that the relationship between endurance exercise and coronary atherosclerosis is non-linear and biologically complex. The strongest signal for increased subclinical disease comes from male veteran endurance athletes with high lifetime exposure, but the prognostic meaning of this disease may not be identical to that in the general population. This apparent paradox—more plaque but often preserved fitness and not necessarily worse outcomes—remains unresolved and underscores the need for further longitudinal studies integrating plaque phenotype, objective training load, ischemia testing, and clinical events.

### 3.3. Silent CAD and Agonistic Versus Non-Agonistic Participation in Master Athletes

A clinically relevant issue in master athletes is that CAD may remain silent or present with attenuated, atypical, or exercise-misinterpreted symptoms. High cardiorespiratory fitness, preserved coronary flow reserve, ischemic preconditioning, and possible collateral adaptation may delay the appearance of typical angina until more advanced disease or higher workloads. In addition, athletes may attribute exertional chest discomfort, dyspnea, fatigue, or reduced performance to musculoskeletal pain, overtraining, respiratory factors, or aging rather than to myocardial ischemia [8]. This may partly explain why anatomical coronary disease may be detected despite normal or non-diagnostic functional testing in selected cohorts, and why CAD may become clinically evident only during very high-intensity exercise or through acute events. Therefore, the absence of typical angina should not be considered sufficient to exclude clinically relevant CAD in master athletes, particularly in the presence of conventional risk factors, abnormal pre-participation screening, reduced performance, or high cumulative training exposure [32].

A further distinction concerns competitive versus non-competitive master athletes. Master athletes are a heterogeneous population including former professional athletes who continue to compete, lifelong competitive endurance athletes, late-onset athletes who begin structured training after adulthood, and highly active recreational individuals. Competitive or agonistic master athletes may be exposed to greater cumulative training load, more frequent high-intensity sessions, competition-related adrenergic surges, dehydration, and a stronger tendency to continue exercising despite symptoms [33]. Conversely, non-competitive or recreationally active master athletes may have lower cumulative exposure and less frequent peak-intensity triggers, although they may also include individuals returning to sport after years of inactivity and therefore with a less favorable cardiovascular risk profile. Current data do not allow a definitive separation of coronary risk according to agonistic versus non-agonistic status, because most studies classify participants by training volume, endurance exposure, age of sport initiation, or athlete-control status rather than by competition level alone. However, the distinction is clinically relevant: in master athletes, sports eligibility and risk interpretation should integrate not only CAC or CCTA findings, but also competition level, lifetime training history, recent training load, symptoms or performance decline, cardiovascular risk factors, and the intended intensity of sport participation [17,33].

## 4. Adaptive Vascular Morphology: Hemodynamic Conditioning of the Coronary Tree

A more coherent way to interpret coronary findings in endurance athletes is to begin not with plaque, but with vascular adaptation. Accordingly, this section separates direct observations in athlete imaging studies from biological mechanisms extrapolated from vascular biology. The latter are presented as plausible pathways rather than established causal mechanisms in endurance athletes. Coronary arteries are load-sensitive biological structures that remodel continuously in response to repeated mechanical stimulation. In endurance athletes, the most immediate and sustained stimulus is hemodynamic. During strenuous exercise, marked increases in heart rate, stroke volume, systolic pressure, pulse pressure, and coronary blood flow expose the arterial wall to repeated episodes of laminar shear stress and cyclic strain [34]. Over years and decades, this cumulative “vascular dose” may induce a conditioned coronary phenotype characterized by outward remodeling, luminal enlargement, altered wall composition, and preserved vasomotor responsiveness [35,36].

### 4.1. Hemodynamic Load as the Initiating Stimulus

During maximal endurance exercise, cardiac output can increase several-fold, often reaching values well above 25 L/min in highly trained athletes. This produces substantial increases in coronary flow velocity, laminar shear stress, and cyclic arterial wall deformation [37]. Endurance athletes therefore expose their coronary arteries to amplified mechanical forces not sporadically, but repeatedly over many years [38,39].

The vascular consequences of endurance exercise are driven less by isolated exercise bouts than by cumulative exposure. Lifetime training volume, intensity, frequency, and duration of athletic career define the hemodynamic burden imposed on the coronary tree. In this sense, endurance training can be understood as a chronic vascular-conditioning stimulus, analogous to how it induces remodeling of cardiac chambers, skeletal muscle, and autonomic control [40].

Although data on discipline-specific coronary phenotypes remain limited, some evidence suggests that the burden and progression of coronary calcification may differ across endurance sports. In the follow-up of the MARC study, cyclists showed a lower incidence and less progression of coronary artery calcification over time than runners and athletes practicing other sports, despite a broadly similar cardiovascular risk profile [41]. This observation raises the possibility that sport-specific hemodynamic and mechanical characteristics may modulate coronary wall stress and, in turn, influence coronary remodeling. Cycling is typically characterized by a more continuous and relatively stable hemodynamic load, whereas running involves repetitive impact-related mechanical stress that may expose the coronary vasculature to greater cyclical strain [42]. Moreover, even in runners, the plaque phenotype described in athlete cohorts is often predominantly calcific, which is generally considered less rupture-prone than mixed or non-calcified plaque [42,43,44]. Importantly, however, these findings should be interpreted cautiously, as current evidence remains observational and does not establish causality.

### 4.2. Mechanotransduction and Endothelial Adaptation

Endothelial cells are the primary mechanosensors of the coronary circulation. Through the glycocalyx, integrins, ion channels, and cytoskeletal structures, they detect changes in shear stress and convert them into intracellular signals. In experimental and non-athlete vascular models, sustained laminar shear stress activates transcriptional programs associated with vascular homeostasis, including Kruppel-like factor 2 (KLF2) signaling and increased endothelial nitric oxide synthase activity [45]. The result is greater nitric oxide bioavailability, enhanced vasodilation, reduced leukocyte adhesion, and suppression of pro-inflammatory and proliferative pathways.

Laminar shear also downregulates NF-κB-dependent inflammatory signaling and reduces expression of adhesion molecules such as VCAM-1 and ICAM-1. These responses promote an anti-atherogenic endothelial phenotype and provide a mechanistic basis for many of the vascular benefits attributed to regular exercise [46]. Yet mechanotransduction is not uniformly protective. Flow patterns vary across the coronary tree, and areas of curvature or bifurcation may experience disturbed or oscillatory flow, creating regional heterogeneity in adaptation [47]. Thus, the same exercise exposure that promotes beneficial remodeling globally may coexist with focal sites of vulnerability.

### 4.3. Adaptive Coronary Remodeling as a Morphological Phenotype

Repetitive high-flow states may promote positive arterial remodeling characterized by luminal enlargement and adaptive wall thickening, in part to normalize wall shear stress despite chronically elevated flow. Changes in extracellular matrix composition, elastin organization, collagen content, and vascular smooth muscle alignment may further contribute to a distinct athlete-specific arterial phenotype [48].

**Figure 3:**
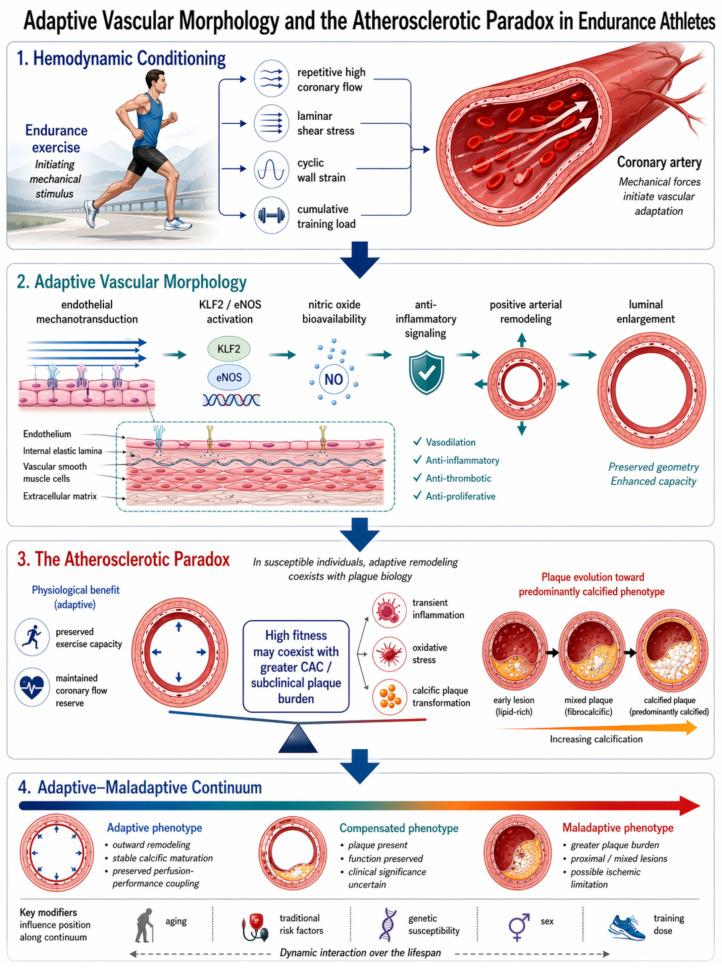
Adaptive vascular morphology and the atherosclerotic paradox in endurance athletes. This schematic illustrates a mechanobiological framework linking long-term endurance exercise to coronary remodeling and plaque phenotype. Panel (**1**) shows the initiating mechanical stimulus acting on the coronary arterial wall. Panel (**2**) depicts adaptive vascular morphology favoring preservation of vascular geometry and flow capacity. Panel (**3**) summarizes the atherosclerotic paradox in susceptible individuals. Panel (**4**) conceptualizes these findings along an adaptive–maladaptive continuum. The position along this continuum is likely modulated over time by aging, conventional cardiovascular risk factors, genetic susceptibility, sex, and cumulative training dose. The clinical imaging associations summarized in this figure are supported by athlete cohort studies, whereas the cellular and molecular mechanisms are extrapolated from experimental vascular biology and should be interpreted as conceptually informative and suggestive of future research directions rather than as definitive evidence of causal mechanisms in endurance athletes. Abbreviations: CAC, coronary artery calcium; CAD, coronary artery disease; CCTA, coronary computed tomography angiography; KLF2, Kruppel-like factor 2; eNOS, endothelial nitric oxide synthase; NO, nitric oxide.

This adaptive morphology is clinically relevant because it may preserve lumen dimensions and perfusion capacity even in the presence of plaque. In other words, the coronary artery in an endurance athlete may not simply accumulate structural change; it may compensate for it. This concept may help explain why, in selected small cohorts, anatomic plaque burden and functional impairment have not always paralleled each other. However, the available evidence remains limited, and preserved exercise capacity should not be assumed to exclude clinically relevant CAD [49,50,51]. Figure 3 illustrates a mechanobiological framework linking long-term endurance exercise to coronary remodeling and plaque phenotype.

## 5. From Adaptive Remodeling to the Atherosclerotic Paradox: Inflammation, Oxidative Stress, and Calcific Plaque Transformation

If adaptive vascular morphology provides the physiological starting point, the atherosclerotic paradox emerges when this adaptive process intersects with plaque biology. A major unresolved question is why some athletes develop greater coronary calcification or plaque burden despite favorable risk profiles and preserved performance. One plausible explanation is that repeated hemodynamic stress, although largely beneficial, may under certain biological conditions be accompanied by transient inflammatory and oxidative responses that influence plaque development and maturation [32,52].

Importantly, these pathways should not be conceptualized as isolated from hemodynamics. They are better understood as downstream or parallel biological responses to the same mechanical environment that drives adaptive remodeling. The paradox, therefore, may not reflect two separate processes—beneficial adaptation on one side and atherosclerosis on the other—but rather a single load-conditioned spectrum in which vascular conditioning and plaque transformation coexist.

### 5.1. Repetitive Inflammatory Activation as a Modifier of Plaque Biology

Habitual physical activity is generally associated with lower chronic systemic inflammation. However, prolonged and intense endurance exercise can acutely increase circulating cytokines, catecholamines, leukocyte trafficking, and other stress-related signals. Acute exercise bouts are accompanied by mobilization of neutrophils, monocytes, and other leukocyte populations, together with short-term elevations in inflammatory cytokines such as tumor necrosis factor-alfa (TNF-α) and several interleukins, including IL-1β, IL-6, IL-8, and IL-10 [53,54,55]. In most athletes, these responses are transient and likely offset by the long-term anti-inflammatory effects of regular training [56]. In susceptible individuals, however, repeated decades-long exposure may create a vascular environment in which endothelial activation, inflammatory-cell recruitment, and local plaque remodeling become more likely.

Repeated inflammatory activation may promote transient endothelial activation, increase expression of adhesion molecules, facilitate leukocyte recruitment into the intima, and amplify local signaling pathways involved in vascular remodeling [57]. In older athletes, or in those with coexisting dyslipidemia, hypertension, prior smoking exposure, or genetic predisposition, such responses may be superimposed on a vessel wall that is already undergoing age-related structural change [58]. Under these circumstances, inflammation may contribute not only to early atherogenic processes, but also to the remodeling of pre-existing plaques. This interpretation remains inferential, but it offers a biologically coherent link between repeated extreme exercise exposure and the paradoxical imaging phenotype described in some master athletes.

### 5.2. Oxidative Stress and Loss of Biological Reserve

A related mechanism involves reactive oxygen species generation during repeated intense exercise. Mitochondrial activation, inflammatory-cell activity, and ischemia–reperfusion-like phenomena may increase oxidative stress and reduce nitric oxide (NO) bioavailability [59]. In principle, this may impair endothelial function, promote LDL oxidation, and facilitate foam-cell formation and low-grade intimal inflammation. These changes could facilitate macrophage recruitment, foam-cell formation, and low-grade intimal inflammation, thereby shifting an otherwise adaptive phenotype toward a more atherosclerotic trajectory. The relevance of oxidative stress in athletes should be interpreted carefully. Moderate habitual exercise enhances endogenous antioxidant defenses and is overall vasoprotective [60]. The question is therefore not whether exercise is intrinsically harmful, but whether repeated exposure to very high exercise loads may, in selected individuals, exceed biological reserve or interact unfavorably with aging and conventional risk factors [61]. In this context, oxidative signaling may function less as an isolated cause of disease than as a permissive pathway that modifies plaque biology when combined with pre-existing susceptibility. This framework is particularly useful because it preserves the well-established cardiovascular benefits of exercise while allowing for the possibility that extreme cumulative exposure may not be biologically neutral in every athlete.

### 5.3. Calcification as Maturation, Healing, or Disease Progression?

The transition from inflammation to calcification is central to the athlete’s paradox. Vascular calcification is now understood as an active and highly regulated biological process involving osteogenic differentiation of vascular smooth muscle cells, extracellular matrix remodeling, and controlled mineral deposition within plaque, rather than passive calcium accumulation alone [62,63]. In endurance athletes, repeated inflammatory and oxidative signaling has been proposed as one mechanism favoring calcific plaque transformation, although direct causal evidence remains limited [64,65].

Exercise-induced changes in mediators of calcium homeostasis may provide an additional mechanistic link. Parathyroid hormone (PTH), a key regulator of calcium and phosphate metabolism, can increase transiently during prolonged or intense endurance exercise, particularly when calcium flux, sweating, and metabolic stress are substantial [66]. Because PTH and related mineral-regulatory pathways are implicated in vascular calcification biology, repeated episodic activation of calcium–phosphate signaling has been proposed as another contributor to calcific plaque remodeling in athletes [67]. Although this hypothesis remains speculative, it is biologically attractive because it links systemic exercise physiology with local vascular biomineralization and may help explain why calcification can emerge even in athletes with otherwise favorable metabolic profiles [68].

**Figure 4:**
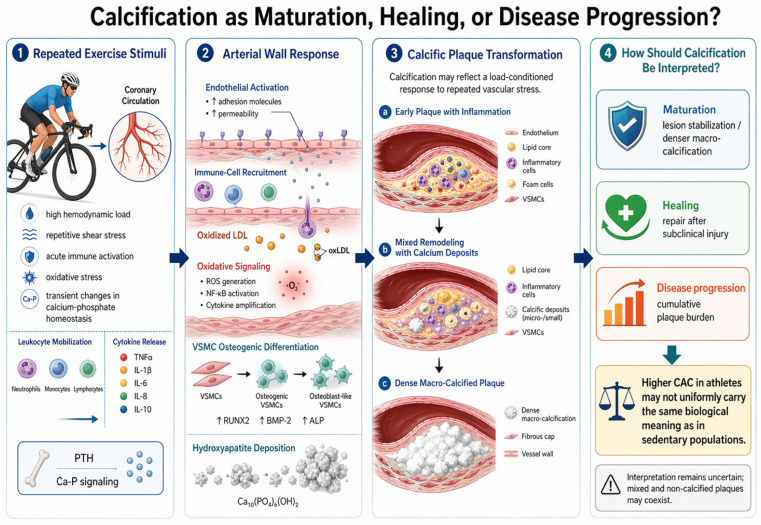
This schematic illustrates how repeated endurance exercise may contribute to calcific plaque transformation through hemodynamic stress, transient inflammation, oxidative stress, and calcium–phosphate signaling. The figure shows progression from early inflammatory plaque to mixed plaque with calcium deposits and dense macro-calcified plaque and highlights that calcification in athletes may reflect lesion maturation, healing after subclinical injury, or cumulative disease progression. The figure also emphasizes that higher coronary artery calcium burden in athletes may have heterogeneous biological correlates, but CAC remains a marker of total plaque burden and should not be assumed to be benign or prognostically neutral. Abbreviations: ALP, alkaline phosphatase; BMP-2, bone morphogenetic protein-2; Ca-P, calcium–phosphate; CAC, coronary artery calcium; eNOS, endothelial nitric oxide synthase; IL, interleukin; LDL, low-density lipoprotein; NF-κB, nuclear factor kappa B; NO, nitric oxide; oxLDL, oxidized low-density lipoprotein; PTH, parathyroid hormone; ROS, reactive oxygen species; RUNX2, runt-related transcription factor 2; TNFα, tumor necrosis factor alpha; VSMCs, vascular smooth muscle cells.

At the tissue level, inflammatory and mineral-regulatory signals may converge on vascular smooth muscle cell phenotype. Chronic or repetitive exposure to cytokine signaling, oxidative stress, and altered calcium–phosphate balance can promote osteogenic pathways, favoring expression of bone-associated proteins and hydroxyapatite deposition within plaque. From this perspective, calcification may represent the morphological endpoint of repeated cycles of vascular stress, subclinical injury, and repair [69]. In some settings, dense macro-calcification may reflect lesion maturation, structural stabilization, or healing after repeated plaque injury. In others, it may simply indicate cumulative plaque burden and advancing disease. Therefore, the interpretation of CAC in athletes should remain clinically cautious. Dense calcification may reflect plaque maturation in some lesions, but a high CAC score also indicates greater total atherosclerotic burden. Athlete status alone should not be used to reclassify elevated CAC as benign, particularly when CAC coexists with mixed or non-calcified plaque, proximal disease, stenosis, symptoms, or conventional risk factors [70]. Figure 4 illustrates how repeated endurance exercise may contribute to calcific plaque transformation.

## 6. The Adaptive–Maladaptive Continuum: Reconciling Coronary Fitness and Coronary Plaque

The concept that best integrates the above observations is an adaptive–maladaptive continuum. At one end lies physiological vascular conditioning: repetitive shear stress promotes endothelial resilience, positive remodeling, preserved vasomotor function, and efficient perfusion under high metabolic demand. In this adaptive window, structural changes may represent optimized coronary morphology rather than clinically important disease [71].

At the other end, the same hemodynamic and biological forces may, under certain conditions, contribute to plaque accumulation, calcification, proximal lesion development, or functionally relevant disease. Aging, dyslipidemia, smoking exposure, hypertension, inflammatory burden, menopause, and inherited susceptibility likely determine where an individual athlete falls along this spectrum [72,73,74].

This model is particularly useful because it reconciles the two apparently contradictory observations that define the field: first, endurance exercise is profoundly cardioprotective; second, selected athletes may still exhibit greater CAC or plaque burden than expected. The paradox, then, may not be a true contradiction but the expression of a load-dependent biological continuum in which adaptation and atherosclerosis are not mutually exclusive [75].

Clinically, this continuum argues against binary labels such as “benign athlete calcification” or “harmful exercise-induced CAD”. Instead, coronary findings should be interpreted in terms of how far remodeling remains compensated, how plaque phenotype evolves, and whether structural change has translated into functional limitation or increased risk [76,77,78,79].

## 7. Sex Differences and Coronary Atherosclerosis in Female Athletes

Sex is an important but still underexplored modifier of exercise-related coronary remodeling. Most studies investigating coronary plaque burden in athletes have included predominantly male cohorts, which limits the generalizability of their findings to women. The currently available data suggest that the relationship between high-volume endurance training and subclinical coronary atherosclerosis differs substantially between sexes [80,81,82].

In contrast to male master athletes, female endurance athletes have generally shown a low prevalence of coronary plaque, minimal CAC burden, and little evidence of exercise-associated acceleration of subclinical atherosclerosis [83,84]. Comparative studies and recent reviews suggest that when coronary atherosclerosis is identified in female athletes, it is more often explained by age and conventional cardiovascular risk factors than by exercise exposure itself [85,86]. These findings are reassuring, but they must be interpreted cautiously because female athlete cohorts have been small and underpowered for definitive conclusions.

Several mechanisms have been proposed to explain this apparent female advantage. Lower circulating androgen levels and higher estradiol levels before menopause may exert protective effects on endothelial function and coronary vasodilation. Female athletes also typically have lower maximal cardiac output and, in many settings, lower cumulative exposure to extreme hemodynamic shear stress than male counterparts. Differences in body composition, exercise intensity, and lifetime training patterns may also contribute.

**Figure 5:**
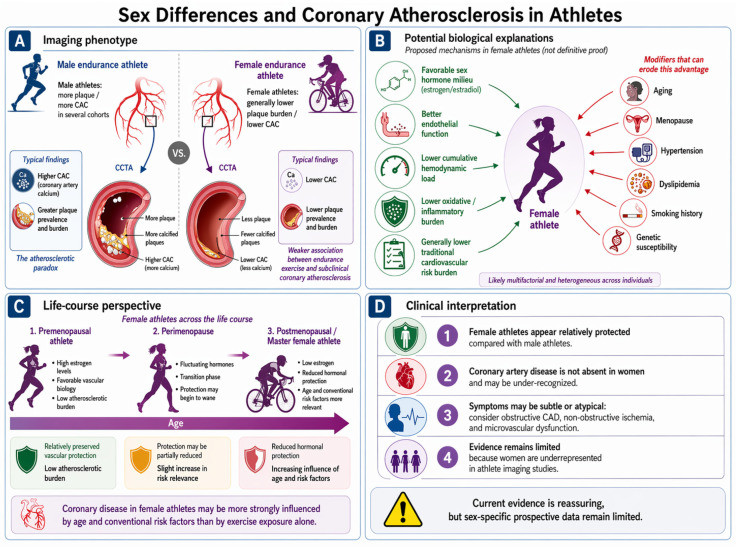
Sex differences and coronary atherosclerosis in athletes. This schematic summarizes current concepts regarding sex-related differences in coronary atherosclerosis in athletic populations. Panel (**A**) illustrates the imaging phenotype, highlighting that male endurance athletes more often show greater CAC burden and higher plaque prevalence in several cohorts than female endurance athletes. Panel (**B**) outlines potential biological explanations for this apparent female advantage. Panel (**C**) presents a life-course perspective, suggesting that coronary protection in female athletes may be greatest before menopause, may begin to decline during the perimenopausal transition, and may become increasingly influenced by age and conventional risk factors in postmenopausal or master female athletes. Panel (**D**) highlights the clinical interpretation: although current evidence is overall reassuring and suggests a lower atherosclerotic burden in female athletes compared with male athletes, coronary artery disease is not absent in women, symptoms may be subtle or atypical, and sex-specific prospective imaging and outcome data remain limited. The mechanisms shown are proposed explanations based on current evidence and should be interpreted as conceptually informative and suggestive of future research directions rather than as definitive evidence. Abbreviations: CAC, coronary artery calcium; CAD, coronary artery disease; CCTA, coronary computed tomography angiography.

Despite these reassuring patterns, coronary disease in female athletes should not be dismissed. Women may present with atypical symptoms, delays in diagnosis, and a broader differential that includes non-obstructive ischemia and microvascular dysfunction. Menopause is particularly relevant because the decline in estrogen exposure may reduce endothelial nitric oxide bioavailability, alter vascular inflammation, worsen lipid profile, and increase central adiposity and blood pressure. Therefore, the coronary phenotype of premenopausal female athletes may differ substantially from that of peri- and postmenopausal master athletes. Future studies should report menopausal status, hormone therapy, reproductive history, and sex-specific risk factors, rather than simply adjusting for sex as a binary covariate. Accordingly, female athletes deserve dedicated prospective investigation rather than extrapolation from male data. Figure 5 summarizes current concepts regarding sex-related differences in coronary atherosclerosis in athletic populations.

## 8. Conclusions

Coronary remodeling in endurance athletes represents a load-conditioned vascular phenotype shaped by the interaction of mechanical stress, mechanotransduction, systemic biology, and time.

The available evidence synthesized in this narrative review suggests that lifelong endurance exercise does not uniformly prevent subclinical coronary atherosclerosis, particularly in older men with high cumulative exposure, though systematic reviews and prospective data are needed to confirm this conclusion. At the same time, coronary disease in athletes often appears phenotypically and functionally distinct from conventional sedentary atherosclerosis, with greater emphasis on calcification, outward remodeling, and preserved exercise capacity.

This perspective argues against binary interpretations. Coronary findings in endurance athletes should be understood along an adaptive–maladaptive continuum, in which structural remodeling may remain physiologically compensated in some individuals while becoming clinically relevant in others. Distinguishing plaque burden from plaque phenotype, and anatomy from function, is central to this interpretation. A mechanobiological framework may therefore offer a useful conceptual explanation for the atherosclerotic paradox in endurance athletes. By integrating exercise dose, endothelial biology, plaque morphology, and physiological performance, it provides a foundation for risk interpretation and more individualized clinical monitoring in the evolving field of sports cardiology. Clinically, particular attention should be given to master athletes with high cumulative exposure or ongoing competitive participation, because CAD may be clinically silent or associated with attenuated symptoms despite relevant anatomical disease. Importantly, this framework should not be interpreted as minimizing the clinical relevance of CAC. Elevated CAC in athletes remains a marker of coronary atherosclerotic burden and should prompt conventional risk-factor optimization and individualized assessment. Future studies should integrate objective training-load assessment, CCTA-based plaque characterization, ischemia testing, sex-specific analyses, and adjudicated clinical outcomes.

## Figures and Tables

**Table 1:** Main athlete-versus-control studies evaluating the association between long-term endurance exercise and coronary atherosclerosis.

Study	Sample: Athletes vs. Controls	Inclusion Criteria	Exclusion Criteria	Age, Years	Matching Variables/Comparator Strategy	Main Findings	Key Limitations
Möhlenkamp et al., 2008 [4]	108 vs. two matched control groups	Apparently healthy male marathon runners aged ≥50 years with ≥5 marathon competitions in the previous 3 years.	Established CVD, DM, angina pectoris, renal failure, musculoskeletal disease preventing future marathon running, psychiatric disease.	57.1 ± 5.6 overall	Two comparators: age-matched controls (8:1) and FRS-matched controls (2:1).	CAC > 100: 36.1% in athletes; 36.3% in age-matched controls; 21.8% in risk factor-matched controls. LGE prevalence 12%; CAC percentile and number of marathons independently predicted LGE. During 2-year follow-up, 4 runners with CAC ≥ 100 had coronary events.	Male-only; self-referred cohort; small number of events.
Schwartz et al., 2014 [18]	50 vs. 23	Men who completed at least 1 marathon yearly for 25 consecutive years.	Contrast allergy, creatinine ≥ 2.0; scans avoided within 2 weeks before/after a marathon	Athletes 59.4 ± 6.7 vs. controls 55.4 ± 10.4	Matched for coronary risk factors	Plaque prevalence was similar, but marathoners had higher total plaque volume, calcified plaque volume, and non-calcified plaque volume; lesion area, diameter stenosis, and lesion length were not significantly different.	Small sample; imperfect matching.
Merghani et al., 2017 [19]	152 vs. 92	Master athletes > 40 years, had run ≥ 10 miles/week or cycled ≥30 miles/week for ≥10 years, and had competed in ≥10 endurance events.	History of CVD, DM, hypertension, hypercholesterolemia, and active or former smokers.	54.4 ± 8.5 overall	Age, sex, and low FRS	Male athletes had more coronary plaques, more multivessel plaques, and 7.5% of male athletes had luminal stenosis ≥50%. Plaques in male athletes were more calcific. No clear excess CAD signal was seen in women. Significant LGE was present in 14.2% of male athletes and 0% of male controls.	Event outcomes unavailable; female subgroup relatively small; highly selected low-risk cohort.
Roberts et al., 2017 (Women) [20]	26 vs. 28	Women who had run ≥1 marathon/year for 10–25 years.	Known CVD; X-ray contrast allergy, serum creatinine > 2.0 mg/dL, pregnancy.	Athletes 56 ± 10 vs. controls 61 ± 10	Propensity match based on self-reported sedentary lifestyle and age.	Calcified coronary plaque was found in 19.2% of female runners vs. 50% of controls; runners had lower lesion prevalence and lower calcified plaque volume.	Small sample; only 5 runners had plaque.
Master@Heart/De Bosscher et al., 2023 [21]	191 vs. 176	Men aged 45–70 years, cycling ≥8 h/week, running ≥6 h/week, or triathlon ≥8 h/week for ≥6 months; lifelong athletes started training <30 years, late-onset athletes > 30 years.	History of CVD; DM; hypercholesterolemia; hypertension; current or past smoking; BMI > 27.2 kg/m^2^; allergy to contrast agents.	55.6 ± 7.8 overall	Analyses adjusted for age, BMI, blood pressure, total and LDL cholesterol, HbA1c, and family history of CAD.	Lifelong athletes had higher odds of ≥1 plaque, ≥1 proximal plaque, ≥1 calcified plaque, ≥1 non-calcified plaque, ≥1 proximal non-calcified plaque, and ≥1 mixed plaque vs. controls. Lifelong athletes had more overall plaque burden, including proximal non-calcified and mixed plaques.	Male-only; white-only cohort; lifetime training load based partly on questionnaires.
Feuchtner et al., 2025 [22]	100 vs. 124	Patients who completed the exercise-habits questionnaire. High-exercise group: training ≥3–5 times/week and ≥1 h/session.	Prior CABG, PCI, ACS, structural/congenital heart disease.	Athletes 56.7 ± 10.9 vs. controls 58.1 ± 10.6	Analyses matched stepwise for sex, family history and smoking, then for all major CV risk factors	No significant difference in CAC, CAD-RADS, obstructive CAD, or MACE over 4 years. No association between high exercise and high-risk plaque or CAC thresholds.	Exercise self-reported; mostly recreational athletes; low event rates.

Abbreviations: ACS, acute coronary syndrome; BMI, body mass index; CABG, coronary artery bypass grafting; CAC, coronary artery calcium; CAD, coronary artery disease; CAD-RADS, Coronary Artery Disease Reporting and Data System; CVD, cardiovascular disease; DM, diabetes mellitus; FRS, Framingham Risk Score; HbA1c, glycated hemoglobin; LDL, low-density lipoprotein; LGE, late gadolinium enhancement; MACE, major adverse cardiovascular events; PCI, percutaneous coronary intervention.

## Data Availability

No new data were created or analyzed in this study.
